# Supplementary material for: A versatile workflow to integrate RNA-seq genomic and transcriptomic data into mechanistic models of signaling pathways
Source: PLoS Comput Biol. 2021 Feb 11;17(2):e1008748. doi: 10.1371/journal.pcbi.1008748 (PMC7904194; doi:10.1371/journal.pcbi.1008748)
Supplement: Attachment — Submitted filename: Point-by-point.docx [file pcbi.1008748.s003.docx]

Point-by-point responses:

Reviewer #1: The manuscript by Garrido-Rodriguez et al., entitled "A versatile workflow to integrate RNA-seq genomic and transcriptomic data into mechanistic models of signaling pathways" provides a novel comprehensive -omics analysis workflow based on containers.

Compared to earlier efforts, novel improvements include the detection and utilization of genomic variances in the RNA-seq data as well as functional annotation for mechanistic pathway analysis. As such, it is potentially of great interest to a wider readership.

The workflow is modeled using the Workflow Description Language, and employs fastp for quality trimming and filtering of reads, FastQC for quality control of read files, as well as STAR, HISAT2, Salmon, FeatureCounts, txImport and edgeR for RNA-seq expression analysis. Normalization applies the standard and recommended Trimmed Means of M-values approach. The Genome Analysis ToolKit and Variant Effect Predictor are used for variant calling and annotation, and HiPathia for functional analysis. Each tool is provided to the user as containerized software via Docker.

COMMENT

=========

While the components are all based on well established methods, the benefits of the proposed approach beyond compiling a range of results -- in a truly integrated analysis -- are not demonstrated in this Research Article. Figures only cover technical issues like runtime and memory use. The authors do not even demonstrate that the claimed lower resource footprint allows the analysis of more demanding sample cohorts.

RESPONSE

=========

Demonstrating the benefits of any integrated analytic approach is not easy because of the lack of a ground truth for functional analysis of multi-omics data. The proposed approach seeks omic data integration in the context of mechanistic interpretation of the results. In order to demonstrate that omic data integration provides more detail and accuracy in the biological interpretation of the results, we have included an example in which the addition of genomic information modifies the results obtained using the transcriptional activity alone, increasing the precision of the estimations of signaling activity. Moreover, the example also addresses the point of testing the workflow on a more “demanding” sample cohort: we have run MIGNON over a set of 462 unrelated human lymphoblastoid cell line samples from the CEU, FIN, GBR, TSI and YRI populations from the 1000 Genomes sample collection. In the new Figure 2, we have depicted how the predicted signaling pathway activities are altered when including the genomic information corresponding to LoF mutations in genes of three different signaling circuits and samples (Figure 2 A and B). In addition, we have explored how, overall, the predicted pathway activities are significantly different when the genomic information is included in the model (Figure 2C, paired Wilcoxon sum-rank test P value < 2.2e-16). Finally, to better explain how the proposed approach works, we have produced a new version of the Figure 1, including a specific step to highlight the integrative aspect of the analysis.

In terms of resource footprint, running MIGNON on the 462 samples from the 1000 genomes project took a total of 101 hours on an HPC environment using the “star” execution mode with the following parameters: Up to 30 workflow-level parallel jobs, 4 threads for fastp, 8 threads for STAR, 6 threads for featureCounts and 1 thread for the rest of tools. This clearly indicates that the workflow can easily scale up to bigger cohorts.

COMMENT

=========

Seeing that the article already provides an overview of earlier well established workflow systems, the key unanswered question must surely be how the new workflow system compares to these in terms of analysis outcomes rather than "workflow system features".

The direct benefits of the presented workflow to others thus remains rather unclear.

RESPONSE

=========

We fully agree with the reviewer that the manuscript would benefit from a comparison of workflows using “analysis outcomes” instead of “workflow system features”. Thus, we have prepared a new table (Table 2) that better illustrates the differences across pipelines.

COMMENT

=========

To better address this, considering that the authors provide no to little justification for their choice of methods, the authors may in revision choose to extend on this and/or provide a demonstration of how easy it would be to exchange a method in the workflow by another.

RESPONSE

=========

Our choice of methods is mainly based on two recent reviews that outline and compare the different approaches to perform the initial analysis of RNA-Seq data (Sahreian, 2017 Nat Comm 8:59 and Stark 2019 Nat Rev Genet 2019 20:631). However, we also agree with the reviewer that some users may want to change a particular tool to perform a specific task (e.g. alignment or quantification). Because of that, we have prepared added a new section to the text, “Modularity of the workflow”, withn the Desing and implementation section, and also added a new section in the documentation (<https://babelomics.github.io/MIGNON/4_advanced.html#modularity>). There explain how users can change one tool from another, using as example a parallel workflow where HT-Seq is used for the gene level quantification instead of featureCounts. The execution of this parallel workflow over example data has also been included in the continuous integration cycle of MIGNON.

COMMENT

=========

While the authors do provide a code repository on github that includes an example script, the provided online documentation is rather basic. The lack of a more comprehensive tutorial or reference limit how easily others could use or build on the presented software.

RESPONSE

=========

We appreciate the observation of the referee on the limitations of the current version of the tutorial. To improve the overall user experience with the software, we have first changed the example data set: instead of using a heavy real dataset, we have prepared a smaller subset with data from a single chromosome, making the test run lighter and thus improving the first contact of the user with the software. In addition, we have encapsulated all the preparation of the test data within a docker container to ensure the reproducibility across different machines. Finally, we have improved the documentation to make it clearer and more detailed (see it at: <https://babelomics.github.io/MIGNON/>). We hope that now the installation of MIGNON is better explained.

COMMENT

=========

Minor: Please revise the manuscript for typos that sometimes make the narrative harder to follow.

RESPONSE

=========

We have reviewed the manuscript and tried to fix all the typos.

COMMENT

=========

Overall, while the reported work is in principle of interest in the field, a number of issues would need to be convincingly addressed to make the manuscript of value in a high-profile journal catering to a wider audience. I encourage the authors to resolve these issues in revision.

RESPONSE

=========

We appreciate the comments made by the referee very much and we have tried to fix the errors and improve the manuscript with the suggestions of the referee. We hope that now the referee finds this new version suitable for publication

Reviewer #2: Review of: A versatile workflow to integrate RNA-seq genomic and transcriptomic data into mechanistic models of signaling pathways

The workflow described in the manuscript is an assembly of widely used alignment algorithms and supporting tools for RNA-seq alignment. The pipelines is implemented using the workflow language WDL and utilized the Cromwell engine for execution.

Additional to RNA-seq alignment, the pipeline templates support variant calling.

The idea of applying variant calling on RNA-seq data is interesting and has not really been utilized broadly. Providing a solution that works without a long software setup is very helpful since several tools need to be applied in series.

Comments:

COMMENT

=========

Calling variants reliably from RNA-seq is not commonly done. This is mainly due to the shallow coverage in most RNA-seq experiments compared to exon sequencing. Furthermore, the depth of reads is directly dependent on the gene expression. What can a user expect in terms of quality of called variants? While a full analysis goes probably too far for this publication, the authors should mention existing studies that analyze this issue and briefly discuss them.

RESPONSE

=========

We are happy to see the interest of the reviewer in the features of our tool and we appreciate very much the positive comments. We agree that the variant calling process for RNA-Seq data should be better discussed in the manuscript. Therefore, we have included two new paragraphs in the introduction and in the design and implementation where we expand information about the known issues in this matter as suggested by the reviewer.

COMMENT

=========

The main contribution of the paper is the example scripts found in the Github repository. While they are mostly self-explanatory, it would be good to discuss each of them more clearly in the help section.

RESPONSE

=========

As requested by the reviewer 1, we have improved the documentation of the tool in the new version of the software. It can be found at the link: https://babelomics.github.io/MIGNON/

COMMENT

=========

When running the example scripts, I ran into several issues related to software version differences compared to the author system setup. For example, the command "gzip -k" command is not supported in many UNIX distributions. Since they are important for system maintenance, they are not easily replaced. (For example, an EC2 instance from AWS does not support this command requiring rewriting). Since the authors are already relying on Dockerized tools, it would be much better to generate Docker images of the example workflows. It should make the execution of the workflow much more seamless.

RESPONSE

=========

We apologize for the issues that some of the scripts might present in specific environments. As explained before as response to one of the comments of reviewer 1, and as suggested by this referee we have prepared a new testing dataset encapsulated into a docker container so now the runMignonExample.sh runs smoothly on different machines.

COMMENT

=========

I also encountered issues with the curl command to download files. This resulted in corrupted files that had to be manually deleted. (I had to switch to wget to make it work, I don't know why). Maybe file downloads can be validated with a checksum if they are provided?

RESPONSE

=========

This is an interesting suggestion, and following it, the new script that prepares the data also checks the integrity of the downloaded data before starting with the runs. Also, as we commented in the previous response, the encapsulation of the download within a docker container improves substantially the user experience when running the example.

COMMENT

=========

The aligners can usually come with a relatively long set of parameters during the index generation step or the alignment step as well, such as k-mer length. I assume the parameters are all default. Is there a way to modify them in the Cromwell JSON file?

RESPONSE

=========

We agree with the reviewer that users should be able of modifying some tool’s input parameters directly at the JSON file. We thank the referee for bringing our attention on this option that would provide an additional flexibility to the workflow and could be used to control specific executions within the pipeline.

Thus, we have included an additional parameter input value that is provided to the tool during the command line execution and that can be included in the input JSON. This option is currently implemented for FastQC, fastp, salmon, HISAT2 and STAR and detailed in the following section of the documentation: https://babelomics.github.io/MIGNON/2_input.html#execution-values
